# Lignocelluloses-Based Furan-Acetone Adducts as Wood Adhesives for Plywood Production

**DOI:** 10.3390/polym15040996

**Published:** 2023-02-16

**Authors:** Lizhen Huang, Wenchang Sun, Li Shuai, Xiaolin Luo, Jing Liu

**Affiliations:** 1 College of Materials Engineering, Fujian Agriculture and Forestry University, Fuzhou 350002, China; 2 National Forestry and Grassland Administration Key Laboratory of Plant Fiber Functional Materials, Fuzhou 350002, China; 3 Jiangsu Provincial Key Laboratory of Pulp and Paper Science and Technology, Nanjing Forestry University, Nanjing 210037, China

**Keywords:** bio-based wood adhesives, aldol condensation, bonding strength, furan-acetone adducts, plywood

## Abstract

Plywood is made of wood veneers that are bonded with adhesives such as urea-formaldehyde, phenol-formaldehyde and melamine-formaldehyde resins. The plywood made from formaldehyde-based adhesives not only releases formaldehyde but also relies on fossil resources. In this article, we synthesized furan-acetone adducts from lignocellulosic biomass in one pot. The furan-acetone adducts could be directly used as adhesives with the addition of phosphoric acid as a curing catalyst. Particularly, with the addition of 5 wt% diphenylmethane diisocyanate (MDI) as a crosslinking agent, both the wet and dry bonding strength of the plywood prepared from the adhesives could meet the minimum requirement of 0.7 MPa (Chinese National Standard GB/T 9846-2015). The possible adhesion mechanism is that the penetration of furan-acetone adhesives into vessels and cell lumens followed by crosslinking during hot-pressing forms mechanical interlocking at the interface of wood veneers, which provides the main bonding strength of plywood. The findings presented here could provide a new way for the efficient preparation of aldehyde-free green wood adhesives and the value-added utilization of woody biomass.

## 1. Introduction

Wood-based panels such as plywood, particleboard, and fiberboard, are often used in furniture and architectural decoration [1]. They are made of wood veneers that are bonded with adhesives such as urea-formaldehyde (UF), phenol-formaldehyde (PF), and melamine-formaldehyde (MF) resins [2]. The global market for wood adhesives is vast, and its total market is expected to reach 21.8 billion dollars by 2028 [3]. Although formaldehyde-based adhesives are good in terms of adhesion properties, processability, quality, and economics, their applications are limited by their dependence on fossil resources and the negative impact of formaldehyde release [4]. With the increasing environmental awareness of human society, research on the synthesis of formaldehyde-free adhesives using renewable biomass has received increasing attention [5], which is expected to reduce the dependence on fossil resources and the emission of formaldehyde [6]. Lignin [7,8,9], tannins [10], proteins [11,12], and starch [13,14] have been evaluated as raw materials to synthesize the substitutes for existing thermosetting adhesives. Similar to starch, polysaccharides such as cellulose and hemicelluloses are renewable feedstocks for the preparation of bio-based wood adhesives [15,16,17,18] due to the advantages of being abundant, inexpensive, and environment-friendly. However, the use of cellulose or hemicelluloses to prepare wood adhesives is rarely reported, mainly because cellulose and hemicelluloses do not show any adhesion properties, even at high temperatures. Therefore, the conversion of cellulose and hemicelluloses into cross-linkable and water-resistant intermediates is a key step in the preparation of wood adhesives.

**Figure 1:**
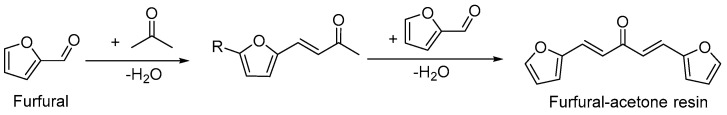
Synthesis of furfural-acetone resin.

Cellulose and hemicellulose derivatives such as furfural and 5-hydroxymethyl (HMF) products could self-condense into humins, but their high permeability into wood cells and water solubility prevents them from forming robust cross-linked adhesives [19]. Instead, furfural-acetone resin, a derivative of furfural and acetone, which has acid and alkali resistance as well as low viscosity, had been widely used as concrete composites, anti-corrosion coatings, laminates, and sand casting, showing good physical and mechanical properties [20]. The synthesis of furfural-acetone resin is generally conducted under alkaline conditions while its curing occurs with acids as catalysts. Furfural-acetone resin can be cured into an insoluble and infusible furan polymer by acidic curing agents (Figure 1).

We believed that the crosslinking ability of furfural-acetone could allow it to be a potential adhesive to replace synthetic resins in the field of wood-based panel production. In the biorefining area, research on the preparation of furan-acetone adducts from biomass-derived platform molecules (such as HMF and furfural) has mainly focused on diesel and jet fuel production [21]. Its synthesis generally starts with furfural or HMF as a feedstock. However, its practical application is limited by the high production cost of furfural and HMF.

The cost-effective conversion of polysaccharides in lignocelluloses to HMF and furfural remains an issue since they tend to further condense to form insoluble humins in an acidic aqueous solution [22]. Therefore, the preparation of low-cost furfural-acetone resin from biomass remains challenging. A few years ago, Shuai and Pan [23] developed a one-pot catalytic reaction system using acetone as a reaction solvent and LiBr and acid as cocatalysts to synthesize furfural-acetone adducts directly from lignocellulosic biomass. In the system, cellulose and hemicelluloses were directly dissolved and hydrolyzed into carbohydrates, which were further converted into furan compounds (i.e., furfural and HMF) with acid and LiBr catalysis. The formed furfural and HMF were immediately captured by acetone molecules to form furan-acetone adducts through aldol condensation. The use of acetone could suppress the self-condensation of furfural and HMF to improve the yield of furan-acetone adducts. Since the furan-acetone adducts have similar structures to the traditional furan-acetone resin, we believe that the furan-acetone adducts could be crosslinked upon heating. In this article, we investigate the potential of biomass-derived furan-acetone adducts as wood adhesives and the curing conditions, and the bonding mechanisms were carefully optimized and analyzed.

## 2. Materials and Methods

### 2.1. Chemicals and Reagents

All reagents were analytically pure and were used immediately upon receipt. Glucose (99%), xylose (99%), tetrahydrofuran (99%), pyridine (99%), glutaraldehyde (50%), epichlorohydrin (98%), hexanediamine (99%), diphenylmethane diisocyanate (98%), and *p*-toluenesulfonic acid (99%) were purchased from Innochem (Shanghai, China). Dichloromethane (99%), lithium bromide (99%), furfural (99%), and phosphoric acid (75 wt%~80 wt%) were purchased from Aladdin^®^ Chemicals (Shanghai, China); hydrochloric acid (37 wt%), acetone (99%), and ethanol (99%) were purchased from China National Pharmaceutical Group Corporation (Beijing, China). 5-hydroxymethylfurfural (HMF) was purchased from Bidepharm (Shanghai, China). Eucalyptus and masson pine (40–80 mesh) were provided by Qingshan Paper Co., Ltd. (Sanming, China), and the straw (40–80 mesh) was obtained from the State Key Laboratory of Bio-based Materials and Green Paper of Qilu University of Technology (Jinan, China).

### 2.2. Synthesis of Carbohydrate-Based Furan-Acetone Adducts

Glucose-based furan-acetone adducts (GluA): glucose (8 g), 8 mL deionized water, 24 g LiBr, 80 mL acetone, and 0.44 mL hydrochloric acid (37 wt%) were added to a 350 mL thick-walled pressure-resistant vessel. After the vessel was sealed, the vessel was heated in an oil bath at 120 °C for 1 h. After the reaction was completed, the residual acetone in the reaction solution was removed by a rotary evaporator, and the solid residue was extracted with 100 mL dichloromethane. After complete dissolution and extraction, the extract was transferred to a centrifuge tube and centrifuged at 10,000 rpm for 15 min, and the upper dichloromethane phase was separated and dichloromethane was removed by a rotary evaporator to obtain a black solid, GluA.

Xylose-based furan-acetone adducts (XylA): The procedure was the same as that for GluA, except that 8 g xylose instead of 8 g glucose was added.

Eucalyptus-based furan-acetone adducts (EA): The procedure was the same as that for GluA, except that 8 g of eucalyptus powder (40−80 mesh) and 0.88 mL of hydrochloric acid (37 wt%) instead of 8 g glucose and 0.44 mL of hydrochloric acid (37 wt%) were added.

Masson pine furan-acetone adducts (MA): The procedure was the same as that for EA except that 8 g of masson pine wood powder (40−80 mesh) was added.

Straw-based furan-acetone adducts (SA): The procedure was the same as that for GluA, except that 8 g of straw powder (40−80 mesh) and 1.02 mL of hydrochloric acid (37 wt%) instead of 8 g glucose and 0.44 mL of hydrochloric acid (37 wt%) were added.

Furfural-acetone adducts: An amount of 0.8 g furfural, 8 mL deionized water, 24 g LiBr, 80 mL acetone, and 0.44 mL hydrochloric acid solution (37 wt%) were added to a 150 mL thick-walled pressure-resistant vessel. After the vessel was sealed, the vessel was heated in an oil bath at 100 °C for 0.5 h. After the reaction was completed, the residual acetone in the reaction solution was removed by a rotary evaporator, and the solid residue was extracted with 100 mL dichloromethane. After complete dissolution and extraction, the extract was transferred to a centrifuge tube and centrifuged at 10,000 rpm for 15 min, the upper dichloromethane phase was separated, and dichloromethane was removed by a rotary evaporator to obtain a black solid (furfural-acetone adducts).

HMF-acetone adducts: An amount of 0.8 g HMF, 8 mL deionized water, 24 g LiBr, 80 mL acetone, and 0.44 mL hydrochloric acid solution (37 wt%) were added to a 150 mL thick-walled pressure-resistant vessel. After the vessel was sealed, the vessel was heated in an oil bath at 120 °C for 1 h. After the reaction was completed, the residual acetone in the reaction solution was removed by a rotary evaporator, and the solid residue was extracted with 100 mL dichloromethane. After complete dissolution and extraction, the extract was transferred to a centrifuge tube and centrifuged at 10,000 rpm for 15 min, the upper dichloromethane phase was separated, and dichloromethane was removed by a rotary evaporator to obtain a black solid (HMF-acetone adducts).

### 2.3. Preparation of Adhesives and Three-Layer Plywood

Preparation of adhesive: Initially, the adducts were vacuum dried at 50 °C for 24 h and then ground into a powder with a quartz mortar. About 10–20 g of the adducts was weighed with the addition of acetone (about 50% mass of the adduct) for dissolving the adduct. The mixture was stirred evenly to form a gel. Next, hydrochloric acid (HCl), phosphoric acid (PA), or *p*-toluenesulfonic acid (*p*-TsOH) was added as a curing agent, and the loading of the curing agent was about 2–15% of the mass of the adduct. Hexamethylene diamine (EDA), glutaraldehyde (GA), epichlorohydrin (ECH), or diphenylmethane diisocyanate (MDI) was added as cross-linking agents during the preparation process, and the amount of the added cross-linking agent was about 3–9% of the adhesive mass. After the addition of all additives, the mixture was fully stirred and then coated on the surface of the veneers.

**Figure 2:**
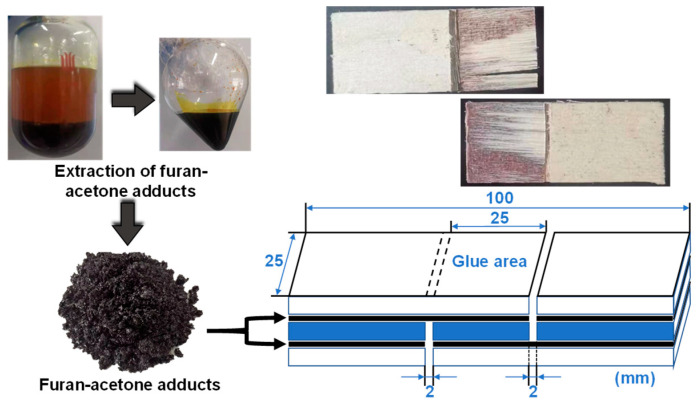
Schematic diagram of plywood specimens used for mechanical strength measurements.

Preparation of three-layer poplar plywood: The poplar veneer was cut into several sheets with a size of 100 mm × 300 mm × 1.6 mm, and the moisture content of the veneers were between 8% and 15%. The grain of each layer was perpendicularly oriented with its adjacent layer. The adhesives were evenly coated on the two sides of the middle-layer veneer (Figure 2) by a rubber brush with a glue sizing of 270 g/m^2^. Prior to hot-pressing, the coated wood veneers were placed in an oven and treated at 80 °C for 8 h to remove excess acetone and prevent glue penetration. Then the three-layer poplar plywood was pressed by a laboratory-scale hot-presser (KS100H, Kesheng Industrial, Dongguan, China) at 120–200 °C for 5–20 min.

### 2.4. Analysis and Characterization of Adhesives

Gas chromatography-mass spectrometry (GC-MS): Ten mg of adduct was dissolved in 1 mL of chloroform in a GC vial. GC-MS analysis was performed using the following temperature program: 40 °C, 8 min; 40–300 °C, 10 °C/min; and 300 °C, 5 min. The chromatographic conditions were as follows: a Techcomp SCION-5MS (Techcomp Instrument Co., Ltd., Shanghai, China) capillary column with high-purity helium as carrier gas was used, and the injection volume, split ratio, and solvent delay time were fixed as 1 μL, 15:1, and 8 min, respectively. The mass spectrometry conditions were as folows: the ion source voltage and its temperature were 70 eV and 200 °C, respectively.

Mechanical analysis of plywood: The plywood samples were stored in air for at least 24 h before mechanical testing. Lap-shear tests of the three-layer plywood were performed on a tensile machine (WDW-200E, Changchun Xinte Testing Machine Co., Ltd., Shenzhen, China) with a crosshead speed of 10 mm/min according to the Chinese National Standard GB/T 9846–2015 [24]. The plywood samples were stored at 25 °C with 50% relative humidity (RH) for at least 24 h before being cut into specimens of a size of 100 mm × 25 mm (Figure 2). After cutting, the specimens were soaked in 63 °C water for 3 h and then cooled at ambient temperature for 10 min before wet strength testing. After soaking, the samples were cooled at ambient temperature (50 ± 2% relative humidity, 25 ± 1 °C) for 10 min and then tested. The reported adhesion results were calculated as the average of measurements of seven specimens.

Observation of glue lines: Nikon optical microscope (Eclipse E200, Nikon Corporation, Nanjing, China) was used to observe the glue lines of the plywood samples. First, slices were cut at the cross-section of the sample using a knife; then, the slices were fixed on a slide; finally, the slide was placed on the optical stage for testing.

Scanning electron microscope (SEM) analysis: SEM (JSM-7500F, Hitachi Limited, Tokyo, Japan) was used to analyze the fracture surface of the glue layer and the morphology of the glue lines of the plywood samples. The samples were firstly dried in a vacuum oven at 50 °C for 24 h. Then, the dried samples were adhered to the sample stage with conductive adhesive and sprayed with gold; finally, SEM tests were performed in high vacuum operation mode at 5.0 kV.

Attenuated Total Reflection-Fourier transform infrared spectroscopy (ATR-FTIR) analysis: ATR-FTIR analysis was carried out on a Nicolet IS 50 spectrometer (Thermo Fisher Scientific Inc., Shanghai, China) over a region of 500–4000 cm^−1^ with 64 scans at a resolution of 4 cm^−1^ at room temperature.

Thermogravimetric (TG) analysis: A thermogravimetric analyzer (Q50, TA Instruments, New Castle, DE, USA) was used to analyze the thermal stability of the cured adhesives. The heating rate was 10 °C/min in a nitrogen atmosphere, the temperature range was 30–800 °C, and the mass of the test sample was 7–10 mg.

## 3. Results and Discussion

### 3.1. Synthesis and Identification of Furan-Acetone Adducts from Biomass

**Figure 3:**
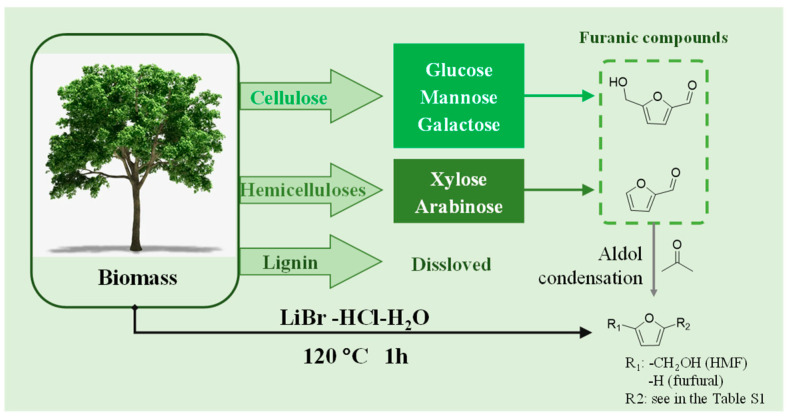
The reaction pathway for preparing furan-acetone adducts directly from lignocellulosic biomass.

The adducts derived from furan and/or acetone were identified by GC-MS. The potential reaction pathways for the self-condensation of acetone molecules and aldol condensation of furfural with acetone molecules were proposed in Figure 3 and Figure S1. The GC-MS spectra of identified products (Figures S2–S4, Supplementary Materials) indicated that other than the aldol condensation of furan compounds with acetone, acetone could self-condense via aldol condensation. Due to the intramolecular dehydration (Figure 3) of acetone self-condensation products, a calculation of n_1_ × 40 + n_2_ × 58 (n_1_, n_2_ = 0, 1, 2…; n_1_ + n_2_ is the number of condensed acetone molecules) was used to estimate the molecular weights of such self-condensation products (Figure S1, Supplementary Materials). Similarly, the calculations of 126 + n_1_ × 40 + n_2_ × 58 and 96 + n_1_ × 40 + n_2_ × 58 could be used to estimate the molecular weights of HMF- and furfural-acetone condensation products, in which n_1_, n_2_ = 0, 1, 2… and n_1_ + n_2_ is the number of condensed acetone molecules. For the acid-catalyzed reactions using lignocellulosic biomass as substrate, the products would be a mixture of acetone self-condensation and HMF- and furfural-acetone adducts. Based on these calculations and the proposed reaction pathways, the structures of these adducts and their molecular weights were presented in Table S1 (Supplementary Materials).

ATR-FT-IR was used to further characterize the biomass-derived furan-acetone adduct samples (Figure S5, Supplementary Materials). Compared with the acetone-acetone adducts, furfural- and HMF-acetone adducts displayed absorptions at 1515 cm^−1^, 1020 cm^−1^, and 966 cm^−1^, indicating that the furan ring was identified in the products and acetone underwent aldol condensation with furan compounds (i.e., furfural and HMF) derived from lignocellulosic biomass. Meanwhile, compared with furfural-acetone adduct and HMF-acetone adduct, GluA, XylA, EA, MA, and SA showed the same absorptions at these three wavenumbers, indicating that furan-acetone adducts could be directly produced from carbohydrates and lignocelluloses in one pot. Compared to the acetone-acetone adducts, GluA, EA, MA, and SA presented a wider absorption band at 3415 cm^−1^, which is a representative of the hydroxyls in the measured samples, indicating the formation of HMF-acetone adducts from these feedstocks. The ATR-FT-IR spectra of furan-acetone adducts combined with the previous GC-MS analysis of these compounds further verified that the furan-acetone compounds were successfully synthesized from lignocellulosic biomass in one pot.

### 3.2. Application of Furan-Acetone Adducts as Adhesives

**Figure 4:**
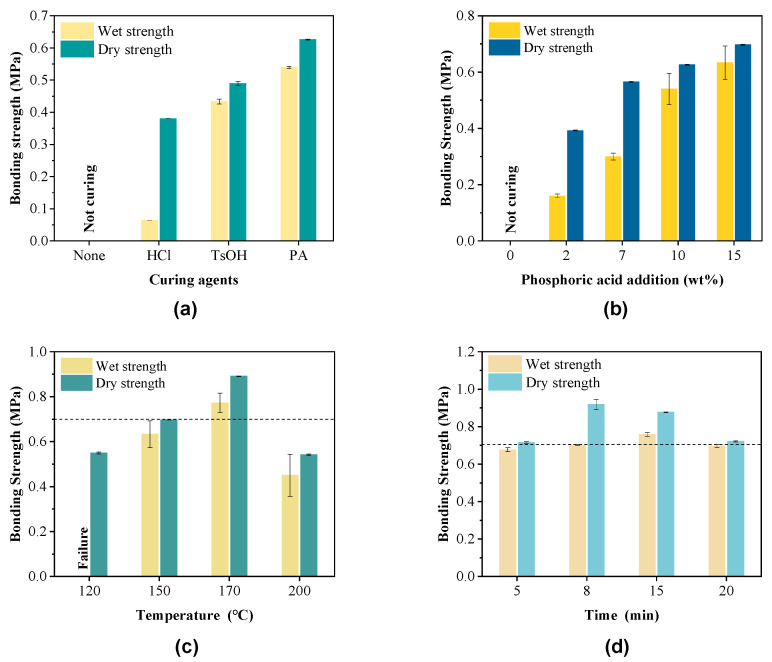
Effects of (**a**) curing agents, (**b**) PA additions, (**c**) pressing temperatures, and (**d**) pressing times on the bonding strength of poplar plywood. The reaction conditions for preparing glucose-derived furan-acetone adducts: 8 g glucose, 8 mL water, 24 g LiBr, 80 mL acetone and 0.44 mL HCl (37 wt%), 120 °C, 1 h. Hot-pressing conditions: 1.0 MPa, 270 g/m^2^, 10 wt% PA, 15 min, 150 °C for (**a**) and (**b**), and 170 °C for (**d**).

The curing of furan-acetone adducts was based on the ring-opening reaction and recondensation of carbonyl groups. Both the ring-opening reaction of the furan ring and the recondensation of carbonyl groups could be accelerated under moderate temperatures with the addition of acids as curing agents. The results in Figure 4a show that PA was mostly effective in promoting the curing of the adhesive and the bonding between the adhesive and the wood veneers, which resulted in the highest wet and dry bonding strengths, i.e., 0.54 MPa and 0.63 MPa. Unexpectedly, the addition of HCl, a strong acid, led to the worst mechanical properties. Such a result could be ascribed to two reasons. First, HCl could be completely ionized in the solvent system to generate hydrogen ions and then accelerated the curing speed of the furan-acetone adhesives. As a result, the cured adhesives could not penetrate effectively the cell lumens and vessels on the surface of wood veneers, which led to a reduction in the mechanical joining effect, the degree of covalent bonding and the hydrogen bonding between the adhesive and the timber; second, HCl was one of the most common strong acids used in industry for biomass pretreatment [25] and it could deconstruct the cell wall structure under high temperatures, thereby reducing the strength of wood veneers. In addition, as a strong inorganic acid, the acidity of *p*-TsOH was between HCl and PA, so its effects on curing adhesive were moderate compared to HCl and PA. Therefore, a medium-strength acid such as PA was suitable for curing furan-acetone adducts at high curing temperatures without deconstructing the structure of wood veneers. With the increase of PA addition (Figure 4b), the strength of the plywood increased. When the PA addition was 10 wt%, the increase in the strength gradually leveled off.

The pressing temperature and time were highly related to the mechanical properties of plywood [26] and were therefore carefully optimized. In general, high temperature is beneficial to accelerate the curing and shorten the curing time [26,27]. When the pressing temperature was increased to 170 °C, both the dry bonding strength (0.87 MPa) and wet bonding strength (0. 76 MPa) (Figure 4c) exceed the minimum requirement of 0.7 MPa of Chinese National Standard GB/T 9846-2015. However, when the temperature rose to 200 °C (Figure 4c), the wet and dry bonding strengths of poplar plywood decreased to 0.45 MPa and 0.54 MPa, respectively. In addition, the pressing time had substantial effects on the strength of plywood (Figure 4d). The dry bonding strength and wet bonding strength of the plywood enhanced 23% and 13% with the extend of pressing time from 5 min to 15 min. However, further extension of the pressing time to 20 min led to the decrease of the dry and wet strengths of the plywood to 0.72 MPa and 0.69 MPa, respectively. It can be seen that a higher temperature in a certain time was conducive to the crosslinking of the adhesive but a high temperature and a prolonged pressing time would result in the over-curing of the furan-acetone adducts, increasing the brittleness of the adhesives and thereby decreasing the strength of the plywood. Additionally, the strong acids as curing agents would accelerate the degradation of the wood veneers at high hot-pressing temperatures, thereby decreasing the strength of the wood veneers [28].

**Figure 5:**
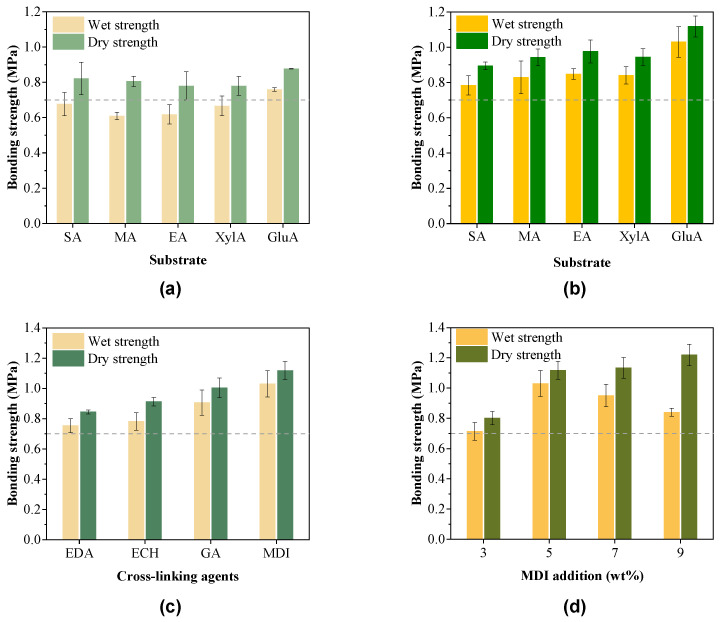
Effects of (**a**) different resin adhesives, (**b**) MDI, (**c**) different cross-linking agents, and (**d**) different MDI additions on the bonding strength of poplar plywood. The reaction conditions: 8 g substrate (e.g., glucose, xylose, eucalyptus, masson pine or straw), 8 mL water, 24 g LiBr, 80 mL acetone, and 0.44 mL HCl (37 wt%) for glucose, xylose, eucalyptus and masson pine, 1.02 mL HCl (37 wt%) for straw, 120 °C, 1 h. Hot-pressing conditions: 170 °C, 1.0 MPa, 270 g/m^2^, 15 min, 10 wt% PA, and 5 wt% MDI.

Based on the optimized conditions above, the poplar plywood prepared from different furan-acetone adducts (i.e., XylA, EA, MA, and SA) were further investigated. All four biomass-based furan-acetone adducts exhibited similar wet and dry bonding strengths (Figure 5a), which were around 0.65 MPa and 0.80 MPa, respectively. Compared with the uncured samples shown in Figure S6a (Supplementary Materials), the cured five furan-acetone adducts showed a different extent of diminished furan C-O-C bond absorption peaks at 1020 cm^−1^ and 966 cm^−1^ as well as diminished furan C-H bond absorption peaks at 879 cm^−1^, 788 cm^−1^, and 1515 cm^−1^, indicating that the furan rings in adhesives were partially opened during the curing process. Simultaneously, the absorption peaks of the carbonyl group at 1714 cm^−1^ were weakened, indicating that the carbonyl groups participated in the curing of the furan-acetone adducts via aldol condensation.

### 3.3. Modification of Furan-Acetone Adhesives

Since the wet strength of the plywood should exceed 0.7 MPa to meet the quality requirement, the effects of several cross-linkers on the strength of plywood prepared from furan-acetone adhesives were analyzed. The cross-linking agents were expected to react with the hydroxyls and/or carbonyls in furan-acetone adducts and hydroxyls on the surface of wood veneers to improve the cross-linking of the furan-acetone adducts and wood-adhesive interface. As shown in Figure 5c, the strength of the plywood after the addition of cross-linking agents was improved, and all of them met the minimum requirement of 0.7 MPa. Among them, the plywood with MDI as a crosslinking agent had the best enhancement effect. This is likely because the isocyanate groups on MDI could easily form carbamate bonds with both the hydroxyl groups on the wood surface and in the furan-acetone adducts during curing, increasing not only the crosslinking of furan-acetone adducts themselves but also the bonding of wood-adhesive interface [29]. Similarly, the aldehyde group in glutaraldehyde (GA) could react with the hydroxyl groups on the wood surface via acetalization and the carbonyl group in furan-acetone adducts to improve the interface crosslinking [30]. Besides, the aldehyde group in glutaraldehyde (GA) could also strengthen the adhesives via aldol condensation of the aldehyde groups of GA and the carbonyls in furan-acetone adducts. The addition of GA could lead to a decent improvement of wet and dry bonding strengths to 0.91 MPa and 1.00 MPa, respectively. Comparatively, the plywood with hexamethylene diamine (EDA) as a cross-linking agent has the worst enhancement effect presumably because the amino group on the EDA is not easily hydroxylated with aliphatic hydroxyl groups and cannot significantly enhance the interfacial crosslinking and hydrogen bonding within the furan-acetone adducts during the curing of the adhesive [31].

Since MDI showed the best promotion effect on the mechanical properties, its addition was further optimized. As shown in Figure 5d, increasing MDI was beneficial to improve the plywood strength from 0.95 MPa to 1.22 MPa. When MDI was increased to 5 wt%, the highest wet adhesive strength of 1.00 MPa was obtained for the plywood. When MDI was further increased to 9 wt%, the wet strength gradually decreased to 0.84 MPa. MDI as a cross-linking agent could introduce hydrophobic groups (benzene rings) into the adhesive to form a more intensely cross-linked network structure, which could effectively prevent the penetration of water molecules and improve the water-resistance of cured adhesives [32].

To further investigate the interaction between the carbohydrate-based furan-acetone adducts and MDI, the samples were analyzed by ATR-FT-IR (Figure S6b, Supplementary Materials). The absorption peaks of -OH at 3415 cm^−1^ and N-H at 1149 cm^−1^ were attenuated for the five samples, which indicated that MDI was indeed introduced into the adhesives, and MDI reacted with hydroxyl groups to form carbamate bonds during the curing process of the adhesives. In addition, compared with the other four furan-acetone adhesives, GluA contained an additional hydroxyl group in the structure of HMF, enabling a higher capability of reacting with isocyanate groups to form cross-linked network and thereby increasing the adhesive strength of the plywood. Therefore, this observation further explained why the glucose-based furan-acetone adducts showed the highest bonding strength of 1.00 MPa for wet bonding strength and 1.12 MPa for dry bonding strength (Figure 5d).

The thermal stability of cured furan-acetone adhesives was investigated using thermogravimetric analysis (TG) (Figure S7, Supplementary Materials). The DTG curves showed that a small weight loss was observed at 125 °C and 145 °C for eucalyptus- and masson pine-based furan-acetone adhesives, respectively, which could be attributed to the evaporation of free and bound water from the adhesives. All of the adhesive samples showed degradation weight loss in the range of 215 to 235 °C. The weight loss was mainly related to the breaking of hydrogen bonds, electrostatic interactions, and some polyurethane and ether bonds. As shown in Table S2 (Supplementary Materials), the degradation temperatures were all higher than those of the samples without the addition of cross-linkers. This result implied that the participation of MDI in the cross-linking of the adhesives through covalent bonds improved the thermal stability of the adhesives. The weight loss in the range of 451−470 °C was mainly due to the degradation of the furan backbone chains in the network structure. Compared with the adducts without the addition of cross-linking agents, the residual carbon rate at 800 °C for all samples increased. The results indicated that the adhesives with the addition of crosslinking agents had higher thermal stability than that without the addition of crosslinking agents. MDI reacted with the carbohydrate-based furan-acetone adducts to form a strong cross-linked network, which improved the thermal stability of the adhesives. Additionally, the residual carbon rate at 800 °C (57.5%) for MDI-modified glucose-derived furan-acetone adhesive was higher than those of other adhesives, indicating that the crosslinking of MDI and C_6_ carbohydrate-derived furan-acetone adducts was denser than that of other adhesives due to the presence of additional hydroxyls in C6 carbohydrate-derived furan-acetone adducts.

The mechanical joining effect, chemical bonding effect, and hydrogen bonding were the main factors influencing the adhesion between the adhesive and the wood veneer. Analysis of the gluing lines of the plywood using an optical microscope showed that the adhesive penetrated the cell lumens and vessels of the poplar wood veneers and filled the cell cavities to form “glue nails” (Figure S8, Supplementary Materials), which enhanced the bonding strength of the plywood [33]. Further analysis of the microscopic morphology of the adhesive layer and the gluing lines using SEM (Figure S9, Supplementary Materials) revealed that the cured MDI-modified furan-acetone adhesive was dense and smooth with the disappearance of microspores compared to the unmodified furan-acetone adducts. The dense network structure could inhibit the penetration of water molecules into the adhesive, thus improving its water resistance and enhancing the adhesive strength of the plywood.

## 4. Conclusions

In conclusion, a variety of carbohydrate-based furan-acetone adduct adhesives from woody biomass were synthesized in a one-pot catalytic reaction system using acetone as a solvent and acidic lithium bromide as a catalyst. This process was carried out under a mild temperature of 120 °C with recyclable catalysts and was able to directly use biomass as feedstock without pretreatment or fractionation. After the optimization of experimental conditions, the best curing effect was obtained with a medium-strength acid, i.e., PA, as a curing catalyst, and the wet and dry bonding strengths could be substantially improved with the addition of crosslinking agents such as MDI. The curing mechanism of furan-acetone adhesives derived from lignocellulosic biomass may mainly involve chemical reactions such as aldol condensation between furan-acetone adducts, the formation of carbamate bonds between side chain hydroxyl groups of furan-acetone adducts, and the hydroxyls on the surface of wood veneers. Based on these observations, the possible adhesion mechanism is that the penetration of furan-acetone adhesives into vessels and cell lumens followed by crosslinking during hot-pressing forms mechanical interlocking at the interface of wood veneers, which provides the main bonding strength of plywood. These sustainable bio-based adhesives can be considered environmentally friendly and cost-effective alternatives to formaldehyde-based adhesives for plywood production. Although carbohydrate-based furan-acetone adhesives could be used to prepare three-layer poplar plywood, the curing conditions of plywood prepared by carbohydrate-based furan-acetone adhesives still need to be optimized to reduce the cost in energy consumption and pressing time compared with those prepared by conventional formaldehyde resin adhesives.

## Data Availability

The research data are included in this paper and its Supplementary Materials.

## Supplementary Materials

The following supporting information can be downloaded at: https://www.mdpi.com/article/10.3390/polym15040996/s1. Figure S1: reaction pathway for the condensation of furfural and one/two molecules of acetone; Figure S2: GC-MS and mass spectra of acetone self-condensation products; Figure S3: (a) GC-MS and mass spectra of furfural-acetone adducts; (b) GC-MS and mass spectra of HMF-acetone adducts; Figure S4: comparisons of the GC-MS spectra of GluA, XylA, EA, MA, and SA; Figure S5: the FT-IR spectra of GluA, XylA, EA, MA, and SA, furfural-acetone adducts, HMF-acetone adducts, and acetone self-condensation adducts without curing; Figure S6: (a) the FT-IR spectra of GluA, XylA, EA, MA, and SA after curing with acid; (b) the FT-IR spectra of GluA, XylA, EA, MA, and SA after acid-catalyzed curing with 5 wt% MDI; Figure S7: (a) the TGA and (b) DTG curves of GluA, XylA, EA, MA and SA derived adhesives that modified by 5 wt% MDI during acid-catalyzed curing process; Figure S8: glue line of poplar plywood prepared by GluA derived adhesives. Reaction condition: 8 g glucose, 8 mL water, 24 g LiBr, 80 mL acetone and 0.44 mL HCl (37 wt%) were reacted at 120 °C for 1 h. Pressing conditions: 170 °C, 1.0 MPa, 270 g/m^2^, 15 min, PA addition 10 wt%, MDI addition 5 wt%; Figure S9: the SEM images of GluA adhesive surface (a) without MDI and (b) with 5 wt% MDI; Table S1: the side chain structure of furan-acetone adducts; Table S2: maximum weight-loss rate temperature and residual carbon rate of furan-acetone adhesives with or without modification by 5 wt% MDI.

## References

[1] Norhazaedawati B., SaifulAzry S.O.A., Lee S.H., Ilyas R.A., Sapuan S.M., Paridah M.T., SaifulAzry S.O.A., Lee S.H. (2022). 4—Wood-Based Panel Industries. Oil Palm Biomass for Composite Panels.

[2] Kristak L., Antov P., Bekhta P., Lubis M.A.R., Iswanto A.H., Reh R., Sedliacik J., Savov V., Taghiyari H.R., Papadopoulos A.N. (2022). Recent Progress in Ultra-Low Formaldehyde Emitting Adhesive Systems and Formaldehyde Scavengers in Wood-Based Panels: A Review. Wood Mater. Sci. Eng..

[3] Hussin M.H., Abd Latif N.H., Hamidon T.S., Idris N.N., Hashim R., Appaturi J.N., Brosse N., Ziegler-Devin I., Chrusiel L., Fatriasari W. (2022). Latest Advancements in High-Performance Bio-Based Wood Adhesives: A Critical Review. J. Mater. Res. Technol.-JMRT.

[4] Arias A., González-Rodríguez S., Vetroni Barros M., Salvador R., de Francisco A.C., Moro Piekarski C., Moreira M.T. (2021). Recent Developments in Bio-Based Adhesives from Renewable Natural Resources. J. Clean. Prod..

[5] Pizzi A. (2016). Wood Products and Green Chemistry. Ann. For. Sci..

[6] Hemmilä V., Adamopoulos S., Karlsson O., Kumar A. (2017). Development of Sustainable Bio-Adhesives for Engineered Wood Panels—A Review. RSC Adv..

[7] Hussin M.H., Samad N.A., Abd Latif N.H., Rozuli N.A., Yusoff S.B., Gambier F., Brosse N. (2018). Production of Oil Palm (Elaeis Guineensis) Fronds Lignin-Derived Non-Toxic Aldehyde for Eco-Friendly Wood Adhesive. Int. J. Biol. Macromol..

[8] Gao S., Liu Y., Wang C., Chu F., Xu F., Zhang D. (2020). Synthesis of Lignin-Based Polyacid Catalyst and Its Utilization to Improve Water Resistance of Urea-Formaldehyde Resins. Polymers.

[9] Domínguez-Robles J., Tarrés Q., Delgado-Aguilar M., Rodríguez A., Espinach F.X., Mutjé P. (2018). Approaching a New Generation of Fiberboards Taking Advantage of Self Lignin as Green Adhesive. Int. J. Biol. Macromol..

[10] Wu H., Liao D., Chen X., Du G., Li T., Essawy H., Pizzi A., Zhou X. (2023). Functionalized Natural Tannins For Preparation of a Novel Non-Isocyanate Polyurea-Based Adhesive. Polym. Test..

[11] Pang H., Ma C., Shen Y., Sun Y., Li J., Zhang S., Cai L., Huang Z. (2021). Novel Bionic Soy Protein-Based Adhesive with Excellent Prepressing Adhesion, Flame Retardancy, and Mildew Resistance. Acs Appl. Mater. Interfaces.

[12] Liu Z., Liu T., Jiang H., Zhang X., Li J., Shi S.Q., Gao Q. (2022). Biomimetic Lignin-Protein Adhesive with Dynamic Covalent/Hydrogen Hybrid Networks Enables High Bonding Performance and Wood-Based Panel Recycling. Int. J. Biol. Macromol..

[13] Sun Y., Gu J., Tan H., Zhang Y., Huo P. (2018). Physicochemical Properties of Starch Adhesives Enhanced by Esterification Modification with Dodecenyl Succinic Anhydride. Int. J. Biol. Macromol..

[14] Sulaiman N.S., Hashim R., Sulaiman O., Nasir M., Amini M.H.M., Hiziroglu S. (2018). Partial Replacement of Urea-Formaldehyde with Modified Oil Palm Starch Based Adhesive to Fabricate Particleboard. Int. J. Adhes. Adhes..

[15] Chrobak J., Iłowska J., Chrobok A. (2022). Formaldehyde-Free Resins for the Wood-Based Panel Industry: Alternatives to Formaldehyde and Novel Hardeners. Molecules.

[16] Khanjanzadeh H., Behrooz R., Bahramifar N., Pinkl S., Gindl-Altmutter W. (2019). Application of Surface Chemical Functionalized Cellulose Nanocrystals to Improve the Performance of UF Adhesives Used in Wood Based Composites—MDF Type. Carbohydr. Polym..

[17] Matsumae T., Horito M., Kurushima N., Yazaki Y. (2019). Development of Bark-Based Adhesives for Plywood: Utilization of Flavonoid Compounds from Bark and Wood. II. J. Wood Sci..

[18] Karagiannidis E., Markessini C., Athanassiadou E. (2020). Micro-Fibrillated Cellulose in Adhesive Systems for the Production of Wood-Based Panels. Molecules.

[19] Coumans F.J.A.G., Overchenko Z., Wiesfeld J.J., Kosinov N., Nakajima K., Hensen E.J.M. (2022). Protection Strategies for the Conversion of Biobased Furanics to Chemical Building Blocks. ACS Sustain. Chem. Eng..

[20] Moses V., Narula A., Chetan N., Mishra R.K. (2022). Hydroxymethyl Furfural (HMF) a High Strength Cellulose Resin for Wood Composite Laminates. Heliyon.

[21] Chheda J.N., Huber G.W., Dumesic J.A. (2007). Liquid-Phase Catalytic Processing of Biomass-Derived Oxygenated Hydrocarbons to Fuels and Chemicals. Angew. Chem. Int. Ed..

[22] Lai F., Yan F., Wang P., Li C., Shen X., Zhang Z. (2021). Efficient Conversion of Carbohydrates and Biomass into Furan Compounds by Chitin/Ag Co-Modified H3PW12O40 Catalysts. J. Clean. Prod..

[23] Shuai L., Pan X. (2016). Method for Producing Liquid Hydrocarbon Fuels Directly from Lignocellulosic Biomass. U.S. Patent.

[24] (2015). Plywood for General Use.

[25] Galbe M., Wallberg O. (2019). Pretreatment for Biorefineries: A Review of Common Methods for Efficient Utilisation of Lignocellulosic Materials. Biotechnol. Biofuels.

[26] Srinivas K., Pandey K.K. (2012). Effect of Heat Treatment on Color Changes, Dimensional Stability, and Mechanical Properties of Wood. J. Wood Chem. Technol..

[27] Rosso L., Negro F., Castro G., Cremonini C., Zanuttini R. (2017). Moisture Dynamics of Thermally Treated Poplar Plywood. Eur. J. Wood Wood Prod..

[28] Lin W.-S., Lee W.-J. (2018). Influence of Curing Temperature on the Bonding Strength of Heat-Treated Plywood Made with Melamine-Urea-Formaldehyde and Phenol-Formaldehyde Resins. Eur. J. Wood Wood Prod..

[29] Sonnenschein M.F., Wendt B.L. (2005). Efficacy of Polymeric MDI/Polyol Mixtures for Binding Wood Boards. Wood Sci. Technol..

[30] Perminova D.A., Malkov V.S., Guschin V., Eisenreich N. (2019). Influence of Glyoxal on Curing of Urea-Formaldehyde Resins. Int. J. Adhes. Adhes..

[31] Hernandez E.D., Bassett A.W., Sadler J.M., La Scala J.J., Stanzione J.F.I. (2016). Synthesis and Characterization of Bio-Based Epoxy Resins Derived from Vanillyl Alcohol. ACS Sustain. Chem. Eng..

[32] Gao Z., Wang W., Zhao Z., Guo M. (2011). Novel Whey Protein-Based Aqueous Polymer-Isocyanate Adhesive for Glulam. J. Appl. Polym. Sci..

[33] Collett B.M. (1972). A Review of Surface and Interfacial Adhesion in Wood Science and Related Fields. Wood Sci. Technol..

