# Supplementary material for: Lignocelluloses-Based Furan-Acetone Adducts as Wood Adhesives for Plywood Production
Source: Polymers (Basel). 2023 Feb 16;15(4):996. doi: 10.3390/polym15040996 (PMC9967313; doi:10.3390/polym15040996)
Supplement: Supplementary file 1 [file polymers-15-00996-s001.zip › polymers-2218593-supplementary.pdf]

*Supplementary Materials*

# Lignocelluloses-Based Furan-Acetone Adducts as Wood Adhesives for Plywood Production

**Lizhen Huang, Wenchang Sun, Li Shuai, Xiaolin Luo and Jing Liu**

**Table S1.** The side chain structure of furan-acetone adducts.

| Number of Acetone Molecules Condensed | $R_2$ [a] | MW [b] of HMF-Acetone Adduct | MW of Furfural-Acetone Adduct |
|---|---|---|---|
| 1 | | 166, 184 | 136, 154 |
| 2 | 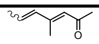 and isomers | 206, 224, 242 | 176, 194, 212 |
| 3 | and isomers | 246, 264 | 216, 234 |
| 4 | 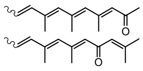 and isomers | 286, 304 | 256, 274 |

[a] Side chain might not be completely dehydrated. [b] Molecular weight.

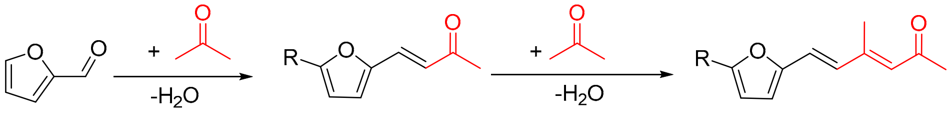


**Figure S1.** Reaction pathway for the condensation of furfural and one/two molecules of acetone.

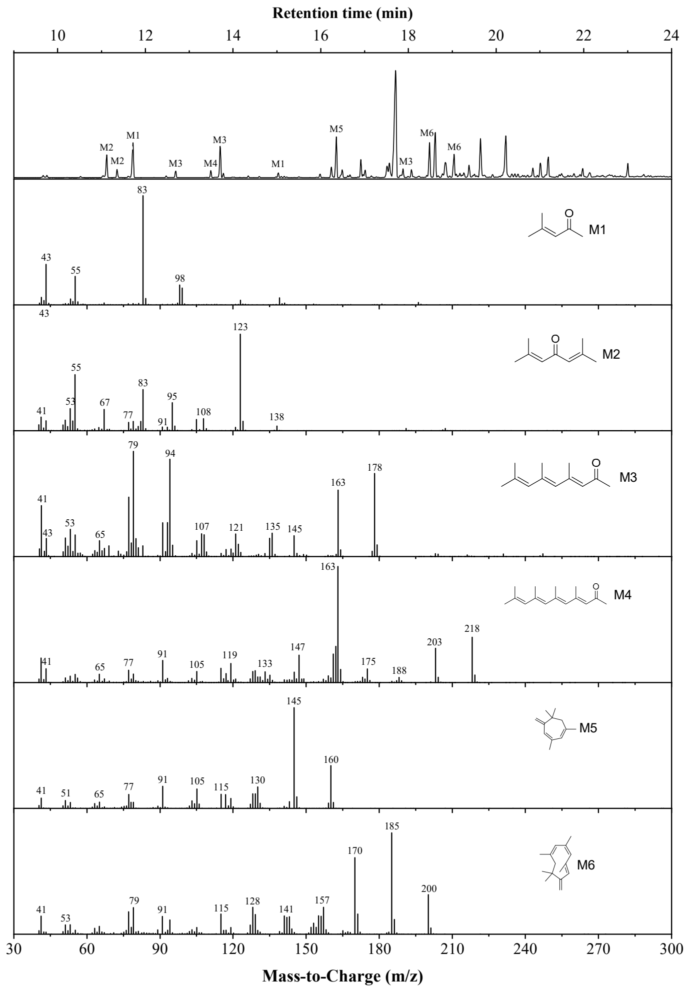


**Figure S2.** GC-MS and mass spectra of acetone self-condensation products.

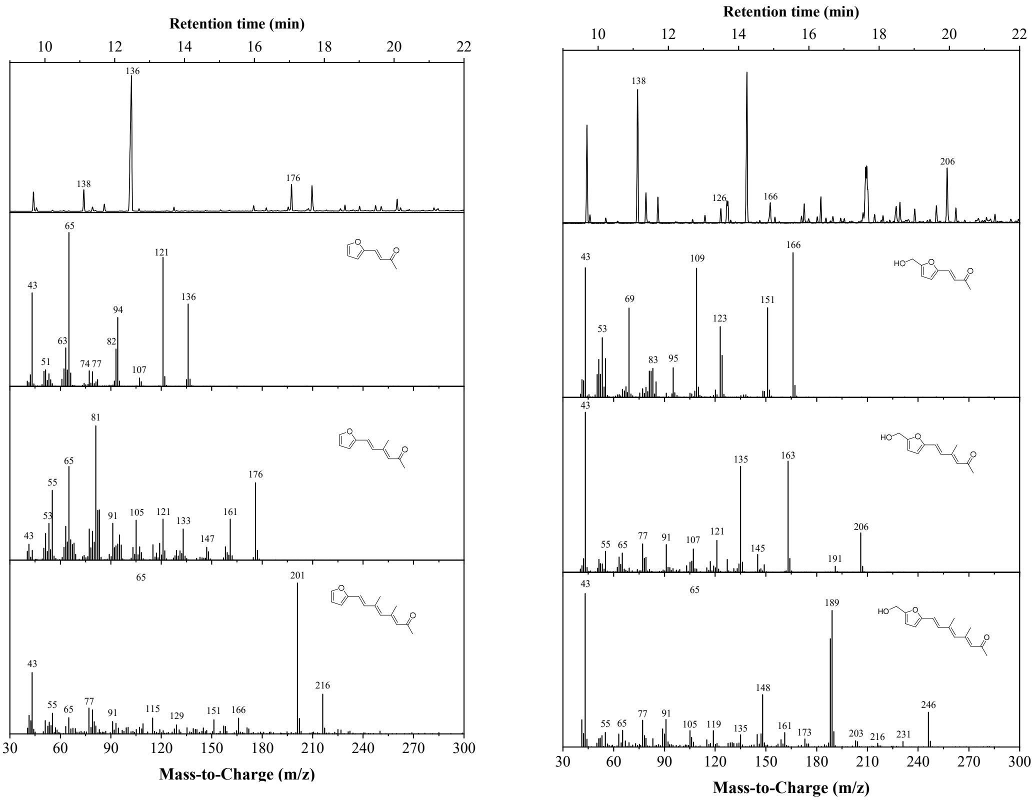


(**a**) (**b**)

**Figure S3.** (a) GC-MS and mass spectra of furfural-acetone adducts; (b) GC-MS and mass spectra of HMF-acetone adducts.

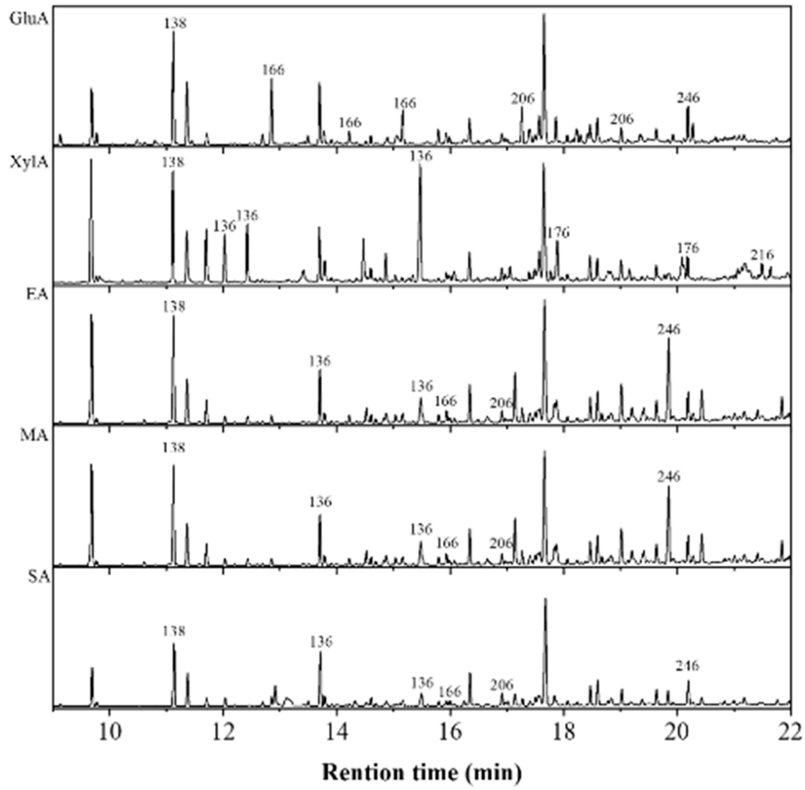


**Figure S4.** Comparisons of the GC-MS of GluA, XylA, EA, MA, and SA.

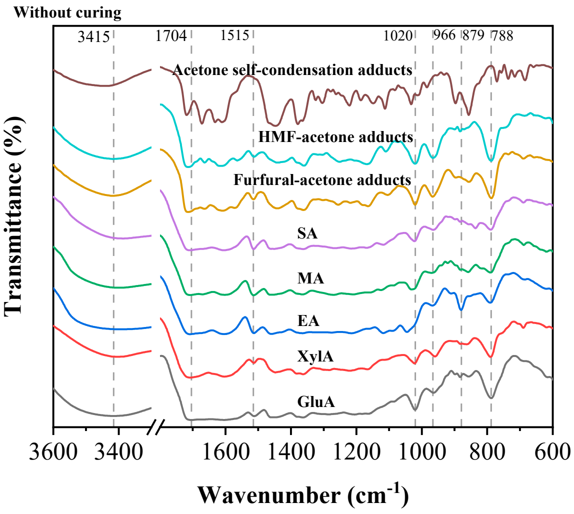


**Figure S5.** The FT-IR spectra of GluA, XylA, EA, MA, and SA, furfural-acetone adducts, HMF-acetone adducts, and acetone self-condensation adducts without curing.

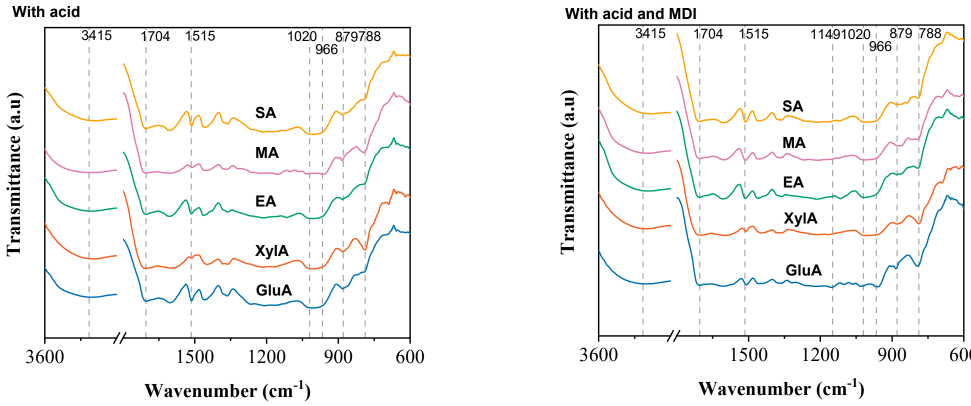


(**a**) (**b**)

**Figure S6.** (a) The FT-IR spectra of GluA, XylA, EA, MA, and SA after curing with acid; (b) the FT-IR spectra of GluA, XylA, EA, MA, SA after acid-catalyzed curing with 5 wt% MDI.

**Table S2.** Maximum weight-loss rate temperature and residual carbon rate of furan-acetone adhesives with or without modification by 5 wt% MDI.

| Sample | $T_1$ (°C) | $T_2$ (°C) | Residual Carbon Rate under 800 °C (%) |
|---|---|---|---|
| GluA | 210 | 454 | 56.8 |
| XylA | 203 | 450 | 53.6 |
| EA | 231 | 449 | 50.8 |
| MA | 230 | 408 | 50.8 |
| SA | 220 | 418 | 45.8 |
| GluA+MDI | 235 | 458 | 57.5 |
| XylA+MDI | 218 | 459 | 55.5 |
| EA+MDI | 229 | 467 | 54.5 |
| MA+MDI | 235 | 470 | 53 |
| SA+MDI | 225 | 451 | 51 |

Note: $T_1$ is the maximum weight loss rate temperature of the first stag, °C； $T_2$: is the maximum weight loss rate temperature of the second stage, °C.

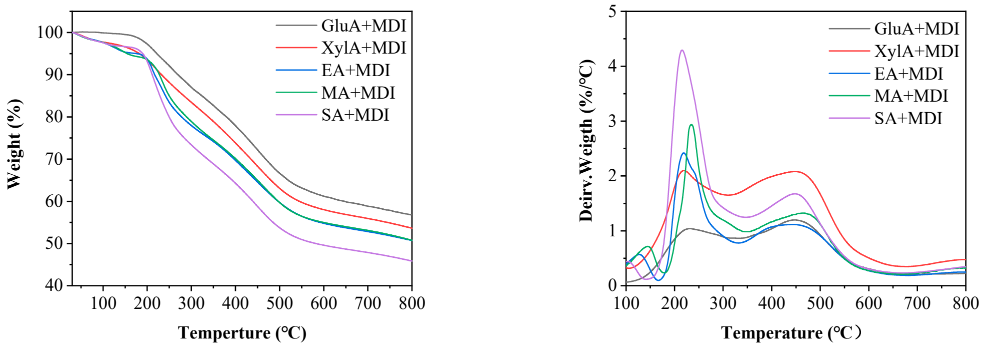


(**a**) (**b**)

**Figure S7.** (a) The TGA and (b) DTG curves of GluA, XylA, EA, MA and SA derived adhesives that modified by 5 wt% MDI during acid-catalyzed curing process.

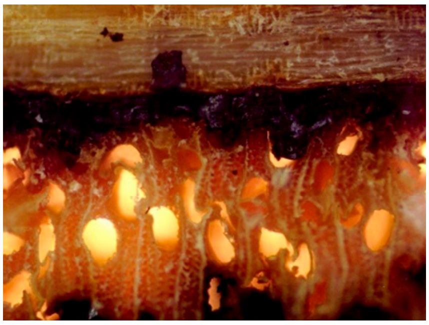

**Figure S8.** Glue line of poplar plywood prepared by GluA derived adhesives. Reaction condition: 8 g glucose, 8 mL water, 24 g LiBr, 80 mL acetone and 0.44 mL HCl (37 wt%) were reacted at 120 °C for 1 h. Pressing conditions: 170 °C, 1.0 MPa, 270 g/m$^2$, 15 min, PA addition 10 wt%, MDI addition 5 wt%.

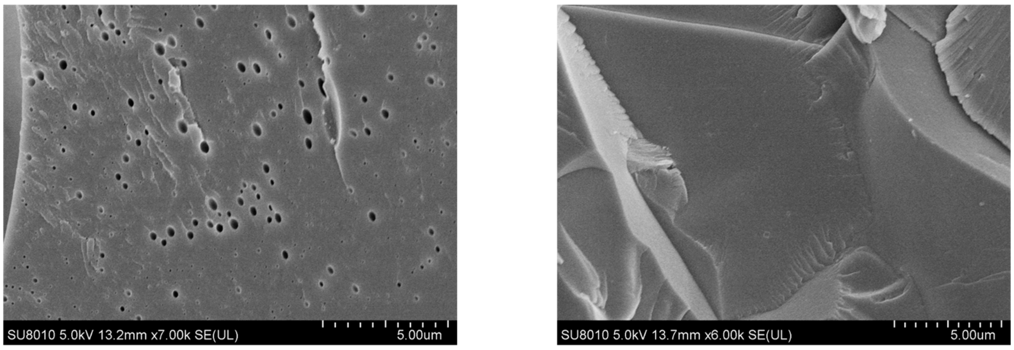


(**a**) (**b**)

**Figure S9.** The SEM images of GluA adhesive surface (a) without MDI and (b) with 5 wt% MDI.
